# Identification of Mutations Related to Cisplatin-Resistance and Prognosis of Patients With Lung Adenocarcinoma

**DOI:** 10.3389/fphar.2020.572627

**Published:** 2020-10-29

**Authors:** Rui Li, Junfang Liu, Zekui Fang, Zhenyu Liang, Xin Chen

**Affiliations:** ^1^ Department of Pulmonary and Critical Care Medicine, Zhujiang Hospital, Southern Medical University, Guangzhou, China; ^2^ Department of Respiratory Medicine, The State Key Laboratory of Respiratory Disease, National Clinical Research Center for Respiratory Disease, Guangzhou Institute of Respiratory Health, First Affiliated Hospital of Guangzhou Medical University, Guangzhou, China

**Keywords:** cisplatin, lung adenocarcinoma, resistance, mutation, chemotherapy

## Abstract

**Background**: Lung adenocarcinoma (LUAD) is the most common histologic type of non-small cell lung cancer (NSCLC; approximately 60%), and platinum-based chemotherapy is the cornerstone of the treatment for patients with LUAD. However, a considerable number of patients experience tumor recurrence after developing cisplatin (*cis*-diamminedichloroplatinum(II) or CDDP) resistance. Therefore, it is particularly important to screen primary CDDP-resistant LUAD patient populations, which can maximize the clinical benefits for these patients.

**Methods**: Data for 61 LUAD cell lines were downloaded from the Genomics of Drug Sensitivity in Cancer (GDSC) database to screen for mutations related to CDDP susceptibility, and we conducted whole-exome sequencing (WES) of tumors from 45 LUAD patients from Zhujiang Hospital of Southern Medical University. Subsequently, the clinical prognostic value of these mutations was verified by using The Cancer Genome Atlas (TCGA)-LUAD cohort and our cohort (*n* = 45).

**Results**: Based on drug sensitivity data for the GDSC-LUAD cell lines and survival analysis of the cohorts TCGA-LUAD and Local-LUAD, we found only one gene (GREB1) with mutations related to decreased CDDP sensitivity as well as worse overall survival (OS) and progression-free survival (PFS) [OS: log-rank *p* = 0.038, hazard ratio (HR; 95% confidence interval (95% CI)): 2.19 (0.73–6.55); PFS: log-rank *p* = 0.001; HR: 4.65, 95% CI: 1.18–18.37]. The GREB1-mutant (GREB1-MT) group had a higher frequency of gene mutations. Additionally, gene set enrichment analysis (GSEA) and single-sample GSEA (ssGSEA) suggested reduced accumulation of intracellular drugs in the GREB1-MT group, in addition to increased drug efflux and enhanced DNA damage repair and intracellular detoxification.

**Conclusion**: This study found that GREB1 mutations may mediate the primary resistance and clinical prognosis of LUAD patients undergoing treatment with CDDP. Further functional analysis showed that GREB1 mutations are related to the known mechanism of CDDP resistance. These results suggest that GREB1 mutations are potential biomarkers for screening of CDDP resistance among LUAD patients.

## Introduction

Lung cancer is the most common malignancy in the world (Bray et al., 2018), and LUAD is the most common histologic type of NSCLC (approximately 60%) (Chalela et al., 2017). Platinum-based chemotherapy is the standard first-line treatment for patients with advanced NSCLC (Jazieh et al., 2017). However, a considerable number of cases relapse after chemotherapy, resulting in a poor prognosis. It has been indicated that chemotherapy cannot eradicate residual cancer cells (Qiu et al., 2019). Moreover, the transcriptional heterogeneity of tumor clones may cause some cases to be resistant to tumor-killing drugs (Stewart et al., 2020). Therefore, it is particularly important to screen for primary CDDP resistance in LUAD patients.

After entering tumor cells, CDDP binds to DNA to induce DNA damage, which is the main cytotoxic mechanism. However, only a small fraction of CDDP enters the cell nucleus to reach DNA (Dolgova et al., 2009), and the remaining CDDP is combined with other biological macromolecules, especially with sulfur-containing molecules, in the cytoplasm, resulting in inactivation (Gibson, 2009). Previous studies on CDDP resistance have mainly focus on drug accumulation, DNA damage repair, key signaling pathways and the tumor microenvironment (TME) (Chen and Chang, 2019). In addition, cancer stem cells play an important role in CDDP resistance and tumor progression (Li et al., 2020; Qiu et al., 2019).

CDDP resistance is dependent of the abnormal regulation of multiple pathways and is the result of multiple factors (Shen et al., 2012; Vandenabeele et al., 2010). Gene mutation is one of the mechanisms of CDDP resistance. p53 mutations increase expression of p53 and bind with the p35 fragment of caspase-9, which inhibits caspase-9 activity and eventually leads to CDDP resistance in cancer (Muller and Vousden, 2014). Furthermore, Sakai et al. (2008) showed that compensatory mutation of BRCA1 and BRCA2 genes can restore the homologous recombination ability of cells, causing them to be more susceptible to CDDP resistance. Similarly, mutations in mismatch repair (MMR) genes (including MLH1 and MSH2) have been suggested to be related to acquired CDDP resistance (Duckett et al., 1996; Vaisman et al., 1998).

Based on the above findings, we sought to determine whether CDDP-resistant LUAD patients have certain genetic mutation characteristics. To this end, we analyzed the expression profiles and CDDP response data of LUAD cell lines in the GDSC database to identify biomarkers for CDDP-resistant LUAD and to explain the related signaling pathways and CDDP resistance mechanisms to provide potential therapeutic strategies for the clinical diagnosis and treatment of LUAD.

## Materials and Methods

### Lung Adenocarcinoma Cell Lines and TCGA-Lung Adenocarcinoma and Local-Lung Adenocarcinoma Cohorts

CDDP response and microarray data for LUAD cell lines were downloaded from the GDSC database. After screening non-synonymous mutations related to CDDP resistance, we analyzed the relationship between non-synonymous mutations (mutation frequency >10%) and CDDP susceptibility. A mutation related to CDDP sensitivity was screened according to the significant difference (*p* value). The unit of measure for the CDDP susceptibility data was the half maximal inhibitory concentration (IC50) value. The GDSC database defines an IC50 value >10 µM as CDDP resistant, whereas an IC50 value <10 µM is considered CDDP sensitive. In addition, we used the “TCGAbiolinks” R package (Colaprico et al., 2016) to download somatic mutation, RNA-seq and clinical data for TCGA-LUAD patients. We retrospectively collected 45 formalin-fixed paraffin-embedded (FFPE) tumor samples with matched germline specimens (*n* = 45) and carried out whole-exome sequencing (WES). The human lung adenocarcinoma tumor specimens, WES, and data processing are detailed in Supplementary Materials.

Kaplan–Meier and log-rank methods were performed to assess the association between mutations and the clinical prognosis of LUAD patients (TCGA-LUAD and Local-LUAD cohorts), which ultimately yielded mutations in GREB1 as associated with sensitivity to CDDP.

### Gene Set Enrichment Analysis and Single-Sample Gene Set Enrichment Analysis

According to the state of GREB1 mutation, analysis of the difference in normalized mRNA data for LUAD cell lines and TCGA-LUAD was carried out using the limma package. The clusterProfiler R package (Yu et al., 2012) was used for GSEA, and the GSVA R package (Hänzelmann et al., 2013) was used for the ssGSEA. The gene sets were obtained from the MSigDB database (Subramanian et al., 2005).

### Statistical Analysis

The webpage visualization tool for the GDSC database was used to display the CDDP sensitivity of pan-cancer cell lines using a box diagram; the Mann-Whitney *U* test was utilized to compare the difference in CDDP susceptibility between wild-type and mutant LUAD cell lines. Fisher's exact test was employed to compare differences in the mutation frequency (top 20) of mutations between wild-type and mutant-type LUAD cell lines/patients (GDSC-LUAD; TCGA-LUAD and Local-LUAD). Kaplan–Meier (KM) analysis involved the log-rank test. A *p* value < 0.05 was considered statistically significant, and all statistical tests were two-tailed. All statistical and visual analyses were carried out in R software (version 3.6.1). In addition, the Complexheatmap (Gu et al., 2016) R package (Version 2.2.0) was used for heatmap visualization, and ggpubr R (Kassambara, 2018) (2.2.0) was used to visualize the boxplot.

## Results

### Sensitivity of Different Tumor Cell Lines and Lung Adenocarcinoma to *cis*-Diamminedichloroplatinum(II)

**FIGURE 1:**
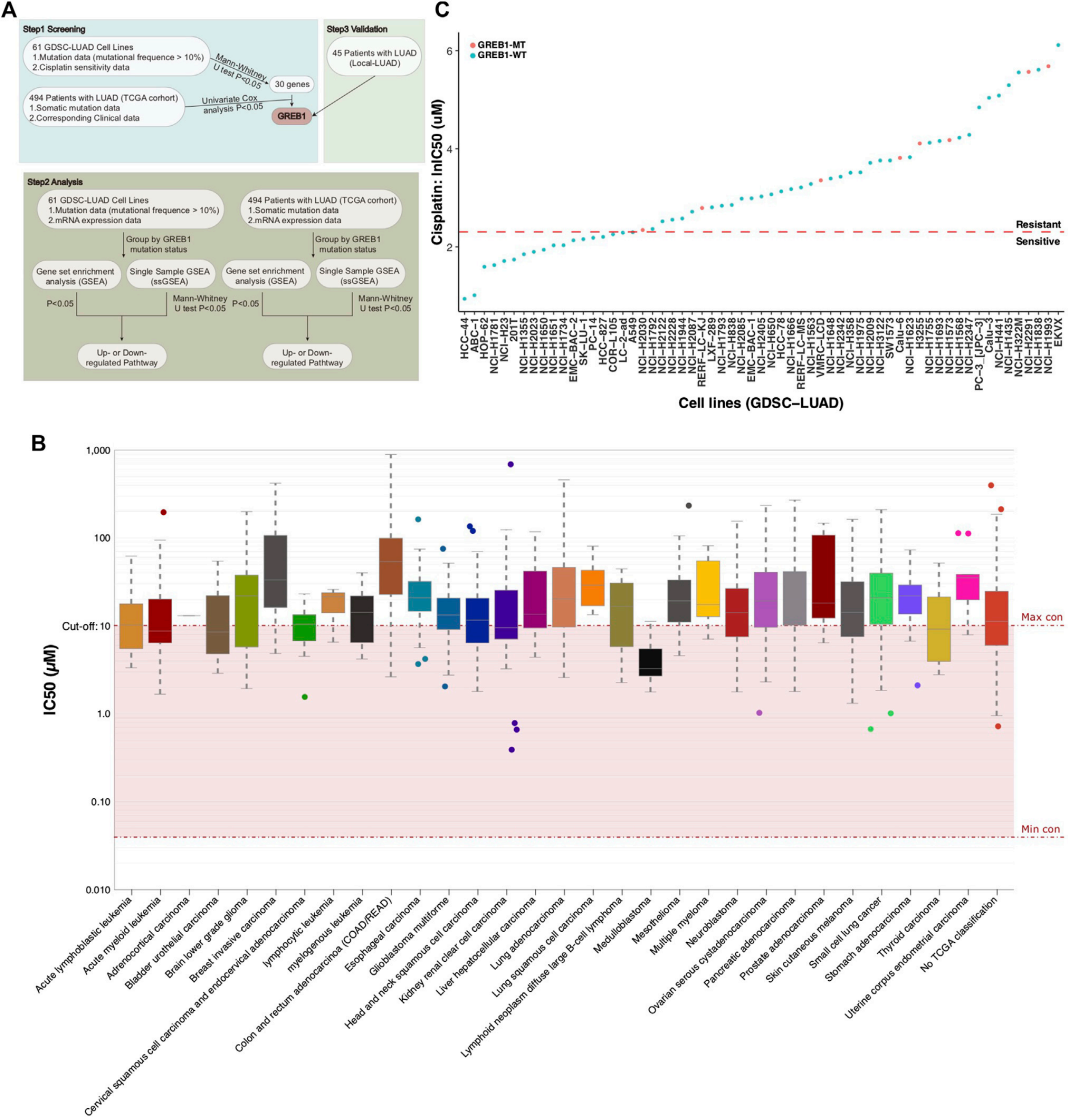
**(A)** The workflow of the bioinformatics analysis. **(B)** The IC50 distribution for CDDP by tissue type. **(C)** Scatter plot of the IC50 distribution for CDDP in sixty-one LUAD cell lines. IC50, half maximal inhibitory concentration; LUAD, lung adenocarcinoma.

As the half maximal inhibitory concentration (IC50) value can effectively distinguish the sensitivity or drug resistance of tumor cell lines to certain drugs (Jazieh et al., 2017; Qiu et al., 2019; Dasari and Tchounwou, 2014, we obtained 62 LUAD cell lines (containing CDDP sensitivity data) from the GDSC database for analysis. CDDP sensitivity, mRNA expression and mutation data were available for 61 LUAD cell lines, and the relevant data were used for downstream analysis (Supplementary Table S1). The detailed analysis flow chart of this study is illustrated in Figure 1A. The prognostic value for LUAD is limited by the mutation rates of genes. We used 10% mutation as a threshold to screen the mutations associated with CDDP sensitivity and employed the Mann-Whitney *U* test to compare the difference in CDDP sensitivity between mutant and wild-type cell lines (mutation rates >10%). We identified thirty mutations significantly associated with the CDDP response (*p* < 0.05). Subsequently, these mutations were used to predict clinical prognosis in the cohort TCGA-LUAD. Finally, mutations significantly associated with CDDP response and survival were validated in the cohort Local-LUAD. Through this process, we only identified GREB1 mutations. Figure 1B shows the IC50 distribution in different tumor tissue samples, showing that most of the cancer cell lines were resistant to CDDP; most of the LUAD cell lines were also CDDP resistant. In total, 18 LUAD cell lines (18/61, 29.5%) were CDDP sensitive and 43 (43/61, 71.5%) CDDP resistant (Figure 1C).

### GREB1 Mutations Are Associated With *cis*-Diamminedichloroplatinum(II) Resistance and Worse Clinical Prognosis

**FIGURE 2:**
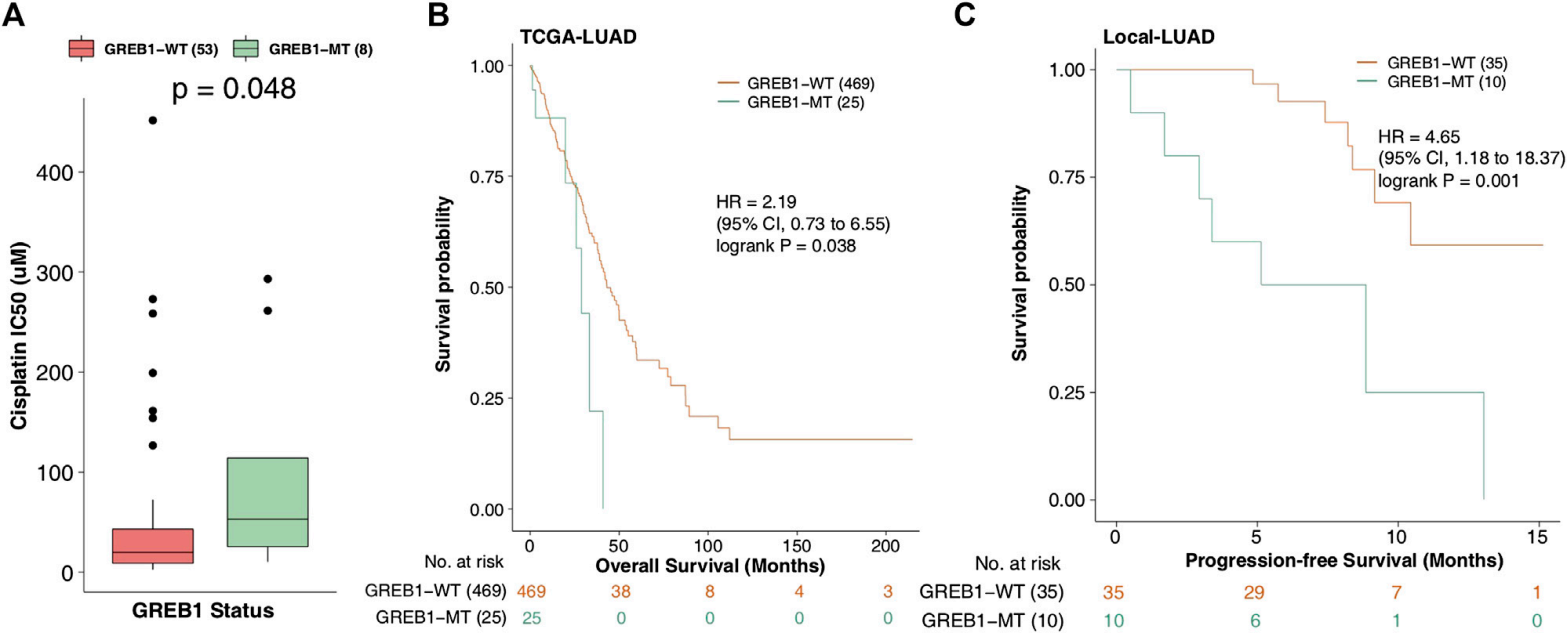
**(A)** IC50 values for CDDP in GDSC-LUAD cell lines with or without GREB1 mutations. **(B)** Regarding OS, the Kaplan-Meier method revealed GREB1 mutations (green) and wild-type GREB1 (orange) for the dataset of 494 patients with LUAD (TCGA database). **(C)** Regarding PFS, the Kaplan-Meier method revealed GREB1 mutations (green) and wild-type GREB1 (orange) in the dataset of 45 patients with LUAD (Local-LUAD cohort). IC50, half maximal inhibitory concentration; CDDP, *cis*-diamminedichloroplatinum(II); GDSC, Genomics of Drug Sensitivity in Cancer; LUAD, lung adenocarcinoma; OS, overall survival; TCGA, The Cancer Genome Atlas; PFS, progression-free survival.

We applied the Mann-Whitney *U* test to compare the difference in CDDP sensitivity between mutant and wild-type cell lines. A total of 30 gene mutations associated with CDDP susceptibility (Supplementary Table S2) were screened. Using the KM method to further analyze the relationship between these mutations and the clinical prognosis of LUAD patients, TCGA-LUAD patients were grouped and analyzed based on the mutation status of these 30 genes. The results showed that only one mutation (GREB1 mutation) was associated with a shorter OS in TCGA-LUAD patients (log-rank *p* = 0.038, HR = 2.19; Figures 2A,B). Next, KM analysis was used to evaluate the association with GREB1 mutations and the prognosis of LUAD, and GREB1-MT was significantly associated with a shorter PFS (log-rank *p* = 0.001; HR: 4.65, 95% CI: 1.18–18.37) in the Local-LUAD cohort (*n* = 45).

### Mutation and Wild-Type GREB1 Mutation Map

**FIGURE 3:**
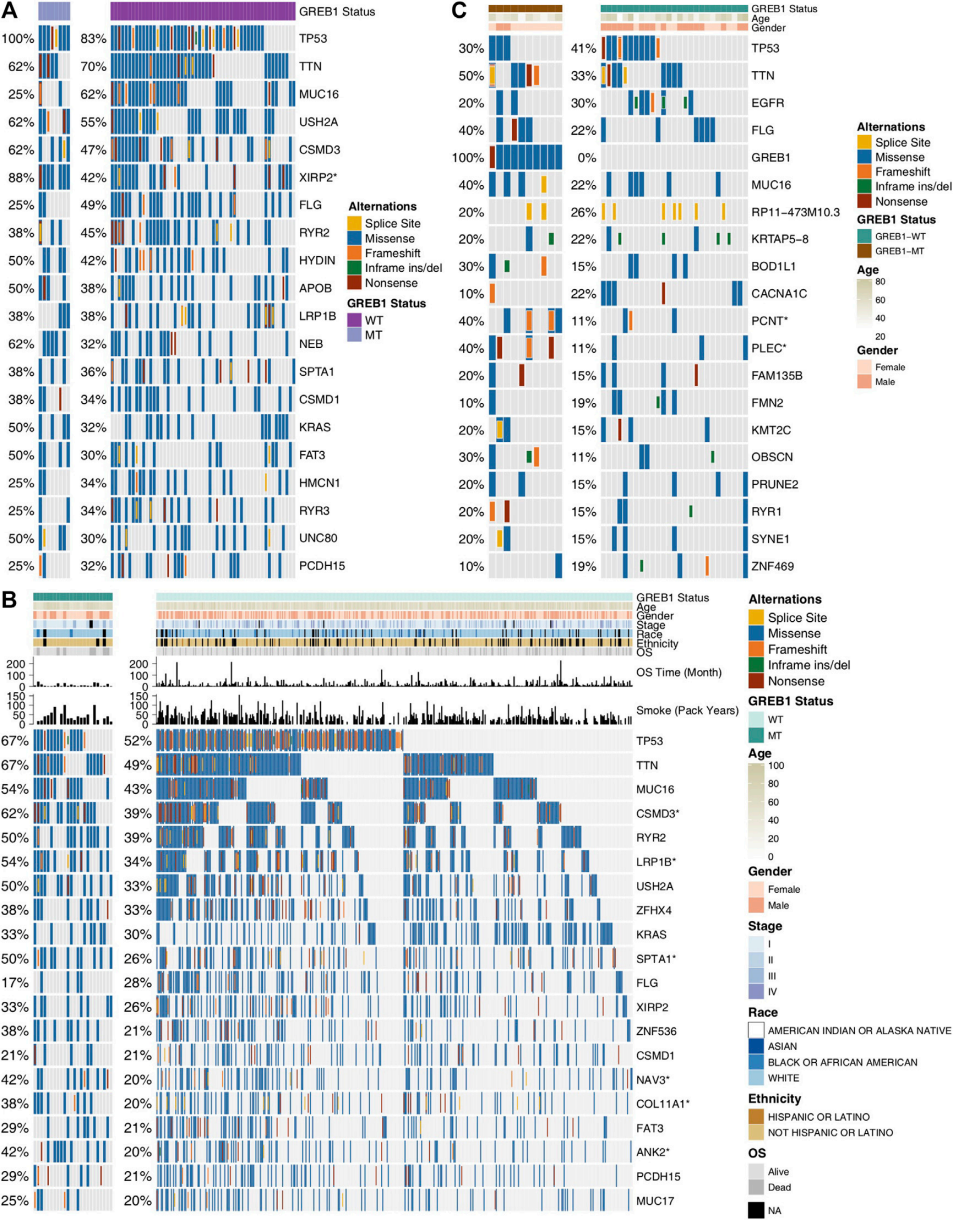
Genomic alterations in LUAD. **(A)** Sixty-one LUAD cell lines are arranged according to their GREB1 mutation status, from left (with GREB1 mutations) to right (without GREB1 mutations). Gene alterations in the LUAD cell line are annotated for each sample according to the color panel below the image. Tumor samples from TCGA-LUAD **(B)** and Local-LUAD **(C)** are arranged according to their GREB1 mutation status, from left (with GREB1 mutations) to right (without GREB1 mutations). Alterations in TCGA-LUAD candidate genes are annotated for each sample according to the color panel below the image. Clinical information for each candidate gene is plotted in the top panel. ****p < 0.0001; ***p < 0.001; **p < 0.01; *p < 0.05 (Fisher’s exact test). LUAD, lung adenocarcinoma; TCGA, The Cancer Genome Atlas.

The 61 LUAD cell lines were grouped according to GREB1 mutation status, and the top 20 mutations of these cell lines are depicted in Figure 3A. The results showed a significantly increased mutation frequency of XIRP2 for GREB1-MT compared to GREB1-WT (7/8, 88%, *p* < 0.05). The mutation frequencies of the remaining 19 genes were not significantly different between GREB1-MT and GREB1-WT. In GDSC cell lines, missense mutation was the main mutation type, whereas in-frame insertion and deletion mutations (indels) had the lowest frequency. Additionally, we analyzed the difference between the mutation frequency of TCGA-LUAD patients between the GREB1-MT and GREB1-WT groups (Figure 3B) and found that the former had higher mutation frequencies in several genes, such as CSMD3 (60% vs. 36%; *p* < 0.05), LRP1B (52% vs. 31%; *p* < 0.05), SPTA1 (48% vs. 24%; *p* < 0.05), NAV3 (40% vs. 19%; *p* < 0.05), COL11A1 (36% vs. 19%; *p* < 0.05), and ANK2 (40% vs. 18%; *p* < 0.05). Missense mutation and in-frame indels were the main and lowest mutation types in TCGA-LUAD patients, respectively. In the Local-LUAD cohort (Figure 3C), the mutation frequencies of several genes were higher in GREB1-MT than in GREB1-WT, such as PCNT (40% vs. 11%; *p* < 0.05) and PLEC (40% vs. 11%; *p* < 0.05).

### GREB1 Mutations Are Related to Abnormal Drug Accumulation, DNA Damage Repair, Extracellular Matrix Synthesis and Other Signal Transduction Pathways

**FIGURE 4:**
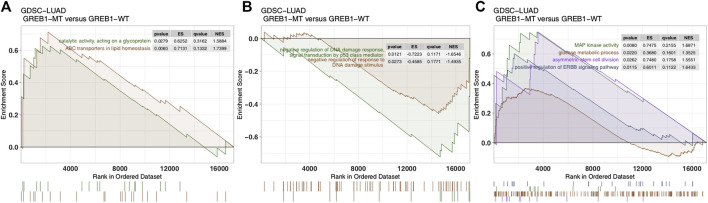
Transcriptome traits in GDSC-LUAD cell lines with or without GREB1 mutations. **(A**–**C)** GSEA of hallmark gene sets downloaded from the MSigDB database. All transcripts were ranked by log2 (fold change) between the GREB1-MT and GREB1-WT LUAD cell lines. Each run was performed with 1,000 permutations. GDSC, Genomics of Drug Sensitivity in Cancer; LUAD, lung adenocarcinoma; GSEA, gene set enrichment analysis; MT, mutant; WT, wild-type.

**FIGURE 5:**
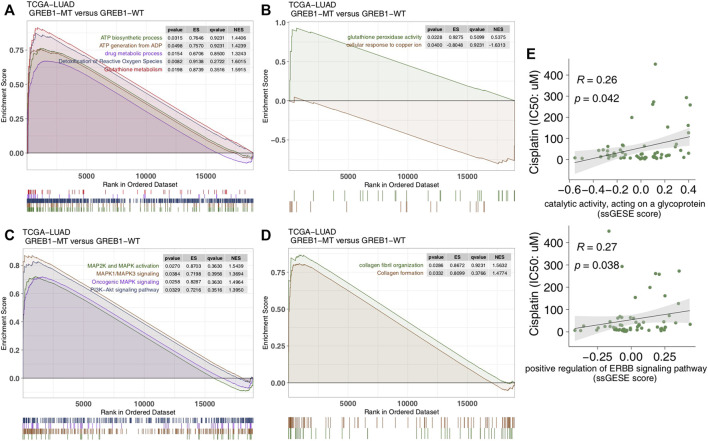
Transcriptome traits in the TCGA-LUAD cohort with or without GREB1 mutations. **(A**–**D)** GSEA of hallmark gene sets downloaded from the MSigDB database. All transcripts were ranked by log2 (fold change) between GREB1-MT and GREB1-WT LUAD patients. Each run was performed with 1,000 permutations. **(E)** Correlation between the single-sample GSEA enrichment score and IC50 values of 55 LUAD cell lines in GDSC (Spearman method). GSEA, gene set enrichment analysis; MT, mutant; WT, wild-type; LUAD, lung adenocarcinoma; IC50, half maximal inhibitory concentration; GDSC, Genomics of Drug Sensitivity in Cancer.

To explore differences in signaling activity between the GREB1-MT and GREB1-WT groups, we used the clusterProfiler R package to search the GSEA database. Figure 4A shows that the GREB1-MT LUAD cell lines were significantly enriched in ATP-binding cassette (ABC) transporters in lipid homeostasis and catalytic activity that act on glycoproteins, suggesting that these tumor cells may exhibit a decrease in uptake and an increase in efflux. Moreover, activities of the DNA damage response, MAP kinase, ErbB signaling and stem cell division and other pathways were significantly upregulated in GREB1-MT LUAD cell lines (Figures 4B,C). Detoxification of reactive oxygen species (ROS) and drug metabolic processes were significantly enriched (Figure 5A) and the cellular response to copper (Cu) ions significantly downregulated in GREB1-MT cell lines (Figure 5B). Additionally, collagen formation and MAPK and PI3K/AKT signaling were significantly enriched in the GREB1-MT group (Figures 5C,D).

We performed ssGSEA on each GDSC-LUAD cell line. The results showed ErbB signaling and catalytic activity acting on a glycoprotein to be positively correlated with CDDP sensitivity (*p* = 0.042, Spearman R = 0.26; *p* = 0.038, Spearman R = 0.27; Figure 5E).

## Discussion

**FIGURE 6:**
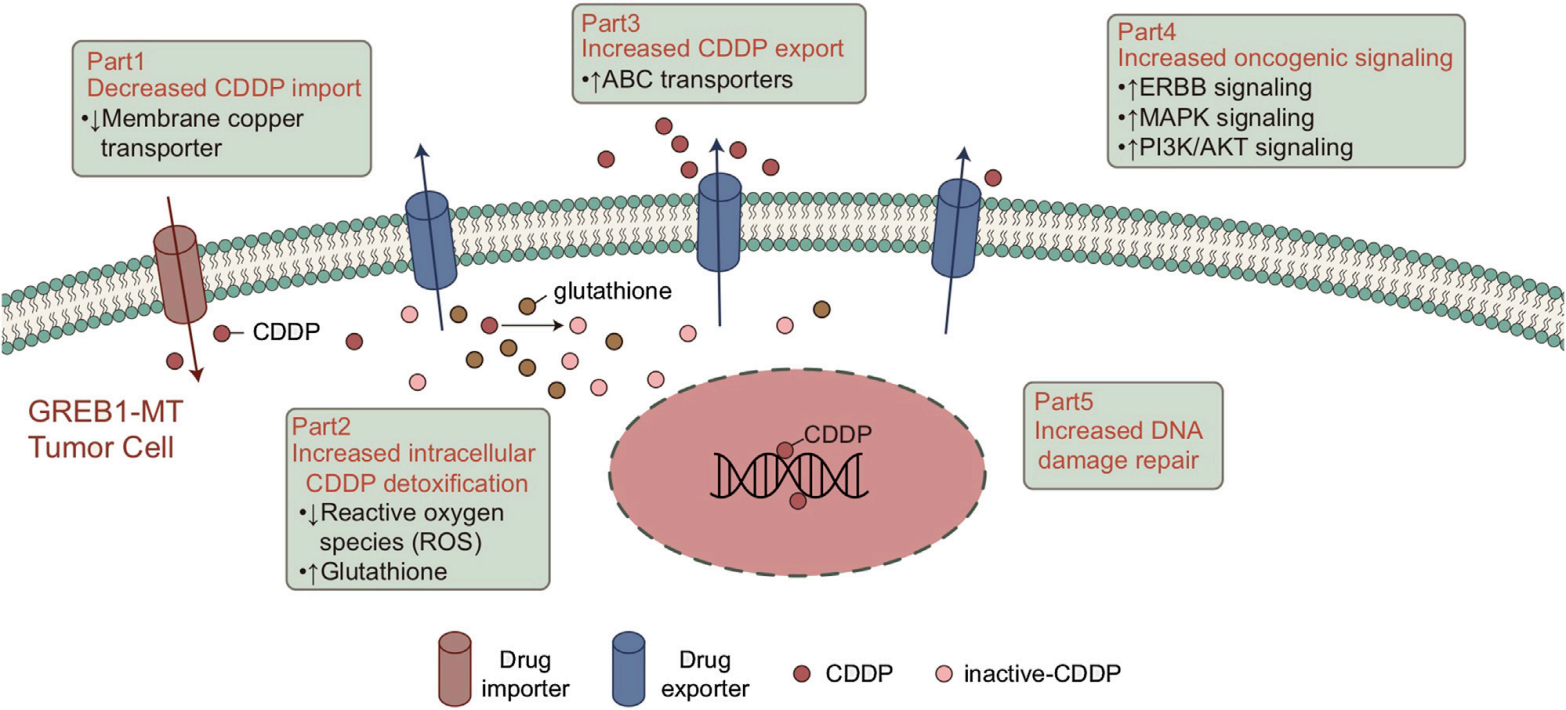
Potential mechanism of GREB1 mutations mediating the resistance of CDDP in LUAD. CDDP, *cis*-diamminedichloroplatinum(II); LUAD, lung adenocarcinoma.

CDDP-based chemotherapy has shown cytotoxicity toward tumor growth in LUAD. Although clinical success has been achieved, the drug resistance of tumor cells has greatly hindered the clinical application of CDDP. Therefore, biomarkers for screening CDDP-resistant LUAD patients are particularly important. In this study, 61 LUAD cell lines with CDDP sensitivity, mRNA expression and mutation data were analyzed. First, we screened for mutations related to CDDP sensitivity. To verify the clinical prognostic significance of these mutations, we analyzed the survival of the cohort TCGA-LUAD based on the mutation status of these genes. The results showed that mutations in only one gene (GREB1) were associated with CDDP resistance and worse clinical outcomes. We used the GSEA and ssGSEA algorithms to evaluate differences in the signaling signature between GREB1-MT and GREB1-WT and attempted to elucidate the mechanism by which GREB1-MT mediates resistance (Figure 6), providing a potential therapeutic strategy for the precise clinical diagnosis and treatment of LUAD.

Tumor cells impair the formation of DNA-platinum adducts by reducing accumulation of the drug in cells, which plays a key role in CDDP resistance. Consistent with previous studies, CDDP binds with target DNA, and expression of CDDP transporters is abnormal in tumor cells, mainly due to downregulation of Cu transporter expression in CDDP uptake. This is responsible for overexpression of the ABC transporter in the efflux of CDDP (Chen and Chang, 2019; Galluzzi et al., 2014; Ohmichi et al., 2005). Our results suggest that in both LUAD cell lines and patients, GREB1-MT is significantly associated with ABC transporters. As ABC transporters significantly reduce the cellular response to Cu ions, GREB1 mutations may mediate CDDP resistance by increasing efflux of CDDP and decreasing its uptake.

In addition to the abnormal regulation of intracellular drug accumulation, the effective detoxification mechanism in cancer cells plays an important role in CDDP tolerance (Chen and Chang, 2019). Mitochondria are the main source of endogenous ROS. Therefore, in the basal state, drug-resistant cells may have a higher ROS level than other tumor cells (Liu et al., 2016), and ROS induced by CDDP can also kill cells (Bray, 2014). Due to the continuous damage caused by ROS, drug-resistant cells establish an effective detoxification mechanism for ROS to ensure that they can survive in a strong oxidizing environment (Bray, 2014; Liu et al., 2016). The pentose phosphate pathway (PPP) is the main antioxidant mechanism in cancer cells and the main source of NADPH. NADPH, which is utilized for the regeneration of reduced GSH and thioredoxin, is involved in neutralizing ROS and maintaining the redox balance. GSH can form a complex with CDDP to avoid protein and DNA damage, leading to CDDP tolerance (Catanzaro et al., 2015). GSEA results suggested significantly increased drug metabolic and GSH biosynthesis processes in the GREB1-MT group, indicating that GREB1 mutations might neutralize ROS by increasing the synthesis of GSH and avoid CDDP-mediated cytotoxicity.

Overall, enhanced DNA damage repair ability can inhibit CDDP cytotoxicity. Indeed, studies have confirmed that enhanced DNA repair activity can limit the CDDP response (Chen and Chang, 2019; Galluzzi et al., 2014). After binding to DNA, most CDDP-induced DNA damage involves the formation of intrastrand DNA-platinum adducts (Dasari and Tchounwou, 2014; Rocha et al., 2018; Ghosh, 2019). The DNA damage repair system is responsible for identifying, verifying, unwinding and removing DNA-platinum adducts and filling the DNA gaps after DNA-platinum adduct clearance (Chen and Chang, 2019). It has been shown that collagen-enriched tumors are associated with decreased sensitivity to CDDP (Senthebane et al., 2018). Additionally, CDDP-induced abnormal changes in triggering signaling pathways after molecular damage may contribute to CDDP resistance (Galluzzi et al., 2014). Wong et al. (2011) showed that inhibition of PI3K, AKT1, and MAPK signaling significantly increased CDDP cytotoxicity in breast carcinoma. The results from the above studies and our analysis suggest that GREB1 mutations may increase activity of the DNA repair system, thereby mediating CDDP tolerance. Additionally, CDDP resistance is mediated by activation of abnormal signaling pathways. GSEA for the cohort TCGA-LUAD suggested that the collagen signature, which is significantly enriched in the GREB1-MT group, may participate in the development of CDDP resistance.

However, there are some limitations in this study. First, our screening of CDDP-resistant mutations was based on LUAD cell lines, and the difference between cell lines and body tissues may cause potential bias in our analysis results. Second, we used only bioinformatics methods to analyze differences in signaling activity between the GREB1-MT and GREB1-WT groups. No cell or animal experiments were used for follow-up verification. Third, the data analysis in this study involved bulk transcriptional datasets, which can only reveal the average level of gene expression in tissues. As the type and status of individual cells were not known, the analysis was unable to uncover the functional status of multiple cell subsets in the tumor. Fourth, the *q*-values of the GSEA were not ideal. Finally, the clinical prognosis of mutations was verified in the cohorts TCGA-LUAD and Local-LUAD. Overall, more samples and prospective studies are needed for further analysis and to validate our findings.

## Conclusion

This study found that GREB1 mutations may mediate primary CDDP resistance and cause a worse clinical prognosis in LUAD patients. Further analysis of the resistance mechanism indicated that GREB1 mutations are associated with abnormal intracellular drug accumulation, DNA damage repair, intracellular detoxification, and abnormal signaling enrichment. These results suggest that GREB1 mutations are potential biomarkers for screening LUAD patients with CDDP resistance.

## Data Availability Statement

According to national legislation/guidelines, specifically the Administrative Regulations of the People’s Republic of China on Human Genetic Resources (http://www.gov.cn/zhengce/content/2019-06/10/content_5398829.htm, http://english.www.gov.cn/policies/latest_releases/2019/06/10/content_281476708945462.htm), no additional data are available at this time. Data of this project can be accessed after an approval application to the China National Genebank (CNGB, https://db.cngb.org/cnsa/). Please refer to https://db.cngb.org/, or email: CNGBdb@cngb.org for detailed application guidance. The accession code can be accessed by emailing the authors. The processed sequencing data can be found here https://github.com/chenxin1020/Cisplatin-LUAD-master.

## Author Contributions

RL, JL, and ZF wrote the manuscript. ZL and XC designed the research. RL, JL, and ZF performed the research. RL, JL, ZF, ZL, and XC writing-review and editing.

## Conflict of Interest

The authors declare that the research was conducted in the absence of any commercial or financial relationships that could be construed as a potential conflict of interest.

## References

[B1] Bray F. Ferlay J. Soerjomataram I. Siegel R. L. Torre L. A. Jemal A. (2018). Global cancer statistics 2018: GLOBOCAN estimates of incidence and mortality worldwide for 36 cancers in 185 countries. Cancer J. Clin. 68 (6), 394–424. 10.3322/caac.21492 30207593

[B2] Bray N. (2014). Lung disease: resetting the redox balance in lung fibrosis. Nat. Rev. Drug Discov. 13 (6), 415 10.1038/nrd4344 24833297

[B3] Catanzaro D. Gaude E. Orso G. Giordano C. Guzzo G. Rasola A. (2015). Inhibition of glucose-6-phosphate dehydrogenase sensitizes cisplatin-resistant cells to death. Oncotarget. 6 (30), 30102–30114. 10.18632/oncotarget.4945 26337086 PMC4745784

[B4] Chalela R. Curull V. Enríquez C. Pijuan L. Bellosillo B. Gea J. (2017). Lung adenocarcinoma: from molecular basis to genome-guided therapy and immunotherapy. J. Thorac. Dis. 9 (7), 2142–2158. 10.21037/jtd.2017.06.20 28840016 PMC5542927

[B5] Chen S.-H. Chang J.-Y. (2019). New insights into mechanisms of cisplatin resistance: from tumor cell to microenvironment. Int. J. Mol. Sci. 20 (17), 4136, 10.3390/ijms20174136 PMC6747329 31450627

[B6] Colaprico A. Silva T. C. Olsen C. Garofano L. Cava C. Garolini D. (2016). TCGAbiolinks: an R/bioconductor package for integrative analysis of TCGA data. Nucleic Acids Res. 44 (8), e71 10.1093/nar/gkv1507 26704973 PMC4856967

[B7] Dasari S. Tchounwou P. B. (2014). Cisplatin in cancer therapy: molecular mechanisms of action. Eur. J. Pharmacol. 740, 364–378. 10.1016/j.ejphar.2014.07.025 25058905 PMC4146684

[B8] Dolgova N. V. Olson D. Lutsenko S. Dmitriev O. Y. (2009). The soluble metal-binding domain of the copper transporter ATP7B binds and detoxifies cisplatin. Biochem. J. 419 (1), 51–56, 3 p following 56 10.1042/BJ20081359 19173677 PMC2825889

[B9] Duckett D. R. Drummond J. T. Murchie A. I. Reardon J. T. Sancar A. Lilley D. M. (1996). Human MutSalpha recognizes damaged DNA base pairs containing O6-methylguanine, O4-methylthymine, or the cisplatin-d(GpG) adduct. Proc. Natl. Acad. Sci. USA. 93 (13), 6443–6447. 10.1073/pnas.93.13.6443 8692834 PMC39042

[B10] Galluzzi L. Vitale I. Michels J. Brenner C. Szabadkai G. Harel-Bellan A. (2014). Systems biology of cisplatin resistance: past, present and future. Cell Death Dis. 5 (5), e1257 10.1038/cddis.2013.428 24874729 PMC4047912

[B11] Ghosh S. (2019). Cisplatin: the first metal based anticancer drug. Bioorg. Chem. 88, 102925 10.1016/j.bioorg.2019.102925 31003078

[B12] Gibson D. (2009). The mechanism of action of platinum anticancer agents—what do we really know about it? Dalton Trans. (48), 10681–10689. 10.1039/b918871c 20023895

[B13] Gu Z. Eils R. Schlesner M. (2016). Complex heatmaps reveal patterns and correlations in multidimensional genomic data. Bioinformatics. 32 (18), 2847–2849. 10.1093/bioinformatics/btw313 27207943

[B14] Hänzelmann S. Castelo R. Guinney J. (2013). GSVA: gene set variation analysis for microarray and RNA-Seq data. BMC Bioinf. 14 (1), 7 10.1186/1471-2105-14-7 PMC3618321 23323831

[B15] Jazieh A. R. Al Kattan K. Bamousa A. Al Olayan A. Abdelwarith A. Ansari J. (2017). Saudi lung cancer management guidelines 2017. Ann. Thorac. Med. 12 (4), 221–246. 10.4103/atm.ATM_92_17 29118855 PMC5656941

[B16] Kassambara A. (2017). ggpubr: “ggplot2” based publication ready plots. CRAN Repository. Available at: https://rpkgs.datanovia.com/ggpubr/index.html (Accessed 5 March, 2020)

[B17] Li M. Lin A. Luo P. Shen W. Xiao D. Gou L. (2020). DNAH10 mutation correlates with cisplatin sensitivity and tumor mutation burden in small-cell lung cancer. Aging 12 (2), 1285–1303. 10.18632/aging.102683 31959735 PMC7053592

[B18] Liu Y. Li Q. Zhou L. Xie N. Nice E. C. Zhang H. (2016). Cancer drug resistance: redox resetting renders a way. Oncotarget 7 (27), 42740–42761. 10.18632/oncotarget.8600 27057637 PMC5173169

[B19] Muller P. A. J. Vousden K. H. (2014). Mutant p53 in cancer: new functions and therapeutic opportunities. Cancer Cell 25 (3), 304–317. 10.1016/j.ccr.2014.01.021 24651012 PMC3970583

[B20] Ohmichi M. Hayakawa J. Tasaka K. Kurachi H. Murata Y. (2005). Mechanisms of platinum drug resistance. TIPS (Trends Pharmacol. Sci.) 26 (3), 113–116. 10.1016/j.tips.2005.01.002 15749154

[B21] Qiu Z. Lin A. Li K. Lin W. Wang Q. Wei T. (2019). A novel mutation panel for predicting etoposide resistance in small-cell lung cancer. Drug Des., Dev. Ther. 13, 2021–2041. 10.2147/DDDT.S205633 PMC6594009 31417239

[B22] Rocha C. R. R. Silva M. M. Quinet A. Cabral-Neto J. B. Menck C. F. M. (2018). DNA repair pathways and cisplatin resistance: an intimate relationship. Clinics 73 (Suppl. 1), e478s 10.6061/clinics/2018/e478s 30208165 PMC6113849

[B23] Sakai W. Swisher E. M. Karlan B. Y. Agarwal M. K. Higgins J. Friedman C. (2008). Secondary mutations as a mechanism of cisplatin resistance in BRCA2-mutated cancers. Nature 451 (7182), 1116–1120. 10.1038/nature06633 18264087 PMC2577037

[B24] Senthebane D. A. Jonker T. Rowe A. Thomford N. E. Munro D. Dandara C. (2018). The role of tumor microenvironment in chemoresistance: 3D extracellular matrices as accomplices. Int. J. Mol. Sci. 19 (10), 2861 10.3390/ijms19102861 PMC6213202 30241395

[B25] Shen D.-W. Pouliot L. M. Hall M. D. Gottesman M. M. (2012). Cisplatin resistance: a cellular self-defense mechanism resulting from multiple epigenetic and genetic changes. Pharmacol. Rev. 64 (3), 706–721. 10.1124/pr.111.005637 22659329 PMC3400836

[B26] Stewart C. A. Gay C. M. Xi Y. Sivajothi S. Sivakamasundari V. Fujimoto J. (2020). Single-cell analyses reveal increased intratumoral heterogeneity after the onset of therapy resistance in small-cell lung cancer. Nat. Can. (Que.) 1 (4), 423–436. 10.1038/s43018-019-0020-z PMC7842382 33521652

[B27] Subramanian A. Tamayo P. Mootha V. K. Mukherjee S. Ebert B. L. Gillette M. A. (2005). Gene set enrichment analysis: a knowledge-based approach for interpreting genome-wide expression profiles. Proc. Natl. Acad. Sci. USA 102 (43), 15545–15550. 10.1073/pnas.0506580102 16199517 PMC1239896

[B28] Vaisman A. Varchenko M. Umar A. Kunkel T. A. Risinger J. I. Barrett J. C. (1998). The role of hMLH1, hMSH3, and hMSH6 defects in cisplatin and oxaliplatin resistance: correlation with replicative bypass of platinum-DNA adducts. Cancer Res. 58 (16), 3579–3585. 9721864

[B29] Vandenabeele P. Galluzzi L. Vanden Berghe T. Kroemer G. (2010). Molecular mechanisms of necroptosis: an ordered cellular explosion. Nat. Rev. Mol. Cell Biol. 11 (10), 700–714. 10.1038/nrm2970 20823910

[B30] Wong S. W. Tiong K. H. Kong W. Y. Yue Y. C. Chua C. H. Lim J. Y. (2011). Rapamycin synergizes cisplatin sensitivity in basal-like breast cancer cells through up-regulation of p73. Breast Cancer Res. Treat. 128 (2), 301–313. 10.1007/s10549-010-1055-0 20686837

[B31] Yu G. Wang L.-G. Han Y. He Q.-Y. (2012). clusterProfiler: an R Package for comparing biological themes among gene clusters. OMICS 16 (5), 284–287. 10.1089/omi.2011.0118 22455463 PMC3339379

