# Supplementary material for: Identification of Mutations Related to Cisplatin-Resistance and Prognosis of Patients With Lung Adenocarcinoma
Source: Front Pharmacol. 2020 Oct 29;11:572627. doi: 10.3389/fphar.2020.572627 (PMC7658917; doi:10.3389/fphar.2020.572627)
Supplement: Supplementary file 1 [file DataSheet1_v1.docx]

**Supplementary Methods**

**Human lung adenocarcinoma tumor specimens.**

The study protocol and informed consent in this study were approved by the Ethics Committee of the Zhujiang Hospital of Southern Medical University, Guangzhou, China. Informed consent in writing was obtained from each patient and the study protocol conformed to the ethical guidelines of the 1975 Declaration of Helsinki as reflected in a prior approval by the Ethics Committee of the Zhujiang Hospital of Southern Medical University, Guangzhou, China. The diagnosis of LUAD was made by pathologists in the Zhujiang Hospital by hematoxylin and eosin (H&E) staining according to histology plus the immunohistochemistry for chromogranin A and synaptophysin. Of the 45 patients, 45 formalin fixed paraffin embedded (FFPE) tumor samples had matched germline specimen (n=45). Whole-exome sequencing (WES) was performed on 45 LUAD FFPE tumor samples and matched germline specimen.

**Whole-exome sequencing and Data processing.**

DNA was extracted from each FFPE specimen (n=45) and matched germline specimen (n=45) using the Gentra Puregene DNA extraction kit (Qiagen) following the protocol of the manufacturer. All sequence data was QC for read counts, quality values, GC-content, and all other relevant parameters with FastQC (v0.10.1). The raw paired sequencing reads of human samples acquired from WES were aligned to the respective human (hg38). The alignment was performed with the BWA aligner(Li and Durbin, 2009) (version 0.6.1-r104). Concordant read-pairs were identified as potential PCR duplicates and were subsequently masked in the alignment file. Additionally, an estimation of human DNA library contamination was implemented to enhance sensitivity and specificity of mutation calling. SAMtools mpileup (0.1.19)(Li et al., 2009) was used to locate non-reference positions in tumor and germline samples. After removal of terminal adaptor sequences and low-quality data, reads were mapped to the reference human genome (hg38) and aligned using BWA (version 0.6.1-r104). MuTect2 (3.4–46-gbc02625)(Cibulskis et al., 2013) was employed to call somatic small insertions and deletions (InDels) and single nucleotide variants (SNVs).

**Reference**

Cibulskis, K., Lawrence, M. S., Carter, S. L., Sivachenko, A., Jaffe, D., Sougnez, C., et al. (2013). Sensitive detection of somatic point mutations in impure and heterogeneous cancer samples. *Nat. Biotechnol.* 31, 213–219. doi:10.1038/nbt.2514.

Li, H., and Durbin, R. (2009). Fast and accurate short read alignment with Burrows-Wheeler transform. *Bioinformatics* 25, 1754–1760. doi:10.1093/bioinformatics/btp324.

Li, H., Handsaker, B., Wysoker, A., Fennell, T., Ruan, J., Homer, N., et al. (2009). The Sequence Alignment/Map format and SAMtools. *Bioinformatics* 25, 2078–2079. doi:10.1093/bioinformatics/btp352.
