# Supplementary material for: Identification of Mutations Related to Cisplatin-Resistance and Prognosis of Patients With Lung Adenocarcinoma
Source: Front Pharmacol. 2020 Oct 29;11:572627. doi: 10.3389/fphar.2020.572627 (PMC7658917; doi:10.3389/fphar.2020.572627)
Supplement: Supplementary file 2 [file Table1_v1.pdf]

Supplementary Table1. The information of sixty-one GDSC-LUAD cell lines.

cell_name	IC50	AUC	Cutoff (uM)	Cisplatin	Whole Exome Sequencing (WES)	Copy Number Alterations (CNA)	Gene Expression	Methylation	Drug Response	Cancer Type	Microsatellite instability Status (MSI)	Screen Medium	Growth Properties
EKVX	451.600283	0.963624	10	Resistant	Y	Y	Y	Y	Y	LUAD	MSS/MSI-L	D/F12	Adherent
NCI-H1993	292.994661	0.980711	10	Resistant	Y	Y	Y	Y	Y	LUAD	MSS/MSI-L	R	Adherent
NCI-H1838	272.860992	0.976749	10	Resistant	Y	Y	Y	Y	Y	LUAD	MSS/MSI-L	R	Adherent
NCI-H2291	261.302163	0.974642	10	Resistant	Y	Y	Y	Y	Y	LUAD	MSS/MSI-L	R	Adherent
NCI-H322M	258.424112	0.973137	10	Resistant	Y	Y	Y	Y	Y	LUAD	MSS/MSI-L	R	Suspension
NCI-H1435	199.198597	0.991629	10	Resistant	Y	Y	Y	Y	Y	LUAD	MSS/MSI-L	D/F12	Semi-Adherent
NCI-H441	161.303315	0.958988	10	Resistant	Y	Y	Y	Y	Y	LUAD	MSS/MSI-L	R	Adherent
Calu-3	154.024697	0.975596	10	Resistant	Y	Y	Y	Y	Y	LUAD	MSS/MSI-L	D/F12	Adherent
PC-3_[JPC-3]	126.672418	0.969022	10	Resistant	Y	Y	Y	N	Y	LUAD	MSS/MSI-L	D/F12	Adherent
NCI-H2347	72.338365	0.962057	10	Resistant	Y	Y	Y	Y	Y	LUAD	MSS/MSI-L	R	Adherent
NCI-H1568	68.258795	0.955115	10	Resistant	Y	Y	Y	Y	Y	LUAD	MSS/MSI-L	R	Adherent
NCI-H1573	65.020981	0.969711	10	Resistant	Y	Y	Y	Y	Y	LUAD	MSS/MSI-L	D/F12	Adherent
NCI-H1693	63.786478	0.92565	10	Resistant	Y	Y	Y	Y	Y	LUAD	MSS/MSI-L	R	Adherent
NCI-H1755	61.619157	0.965047	10	Resistant	Y	Y	Y	Y	Y	LUAD	MSS/MSI-L	R	Adherent
H3255	60.73665	0.955985	10	Resistant	Y	Y	Y	Y	Y	LUAD	MSS/MSI-L	R	Adherent
NCI-H1623	45.830496	0.94482	10	Resistant	Y	Y	Y	Y	Y	LUAD	MSS/MSI-L	D/F12	Adherent
Calu-6	45.236764	0.950445	10	Resistant	Y	Y	Y	Y	Y	LUAD	MSS/MSI-L	D/F12	Adherent
SW1573	42.948395	0.932835	10	Resistant	Y	Y	Y	Y	Y	LUAD	MSS/MSI-L	D/F12	Adherent
NCI-H3122	42.922853	0.953505	10	Resistant	Y	Y	Y	Y	Y	LUAD	MSS/MSI-L	R	Adherent
NCI-H2009	40.843076	0.950771	10	Resistant	Y	Y	Y	Y	Y	LUAD	MSS/MSI-L	D/F12	Adherent
NCI-H1975	33.644183	0.932949	10	Resistant	Y	Y	Y	Y	Y	LUAD	MSS/MSI-L	R	Adherent
NCI-H358	33.407376	0.936939	10	Resistant	Y	Y	Y	Y	Y	LUAD	MSS/MSI-L	R	Adherent
NCI-H2342	30.828497	0.938782	10	Resistant	Y	Y	Y	Y	Y	LUAD	MSS/MSI-L	D/F12	Adherent
NCI-H1648	29.689948	0.923848	10	Resistant	Y	Y	Y	Y	Y	LUAD	MSS/MSI-L	D/F12	Adherent
VMRC-LCD	28.607681	0.972163	10	Resistant	Y	Y	Y	N	Y	LUAD		D/F12	Adherent
NCI-H1563	26.574865	0.920741	10	Resistant	Y	Y	Y	Y	Y	LUAD	MSS/MSI-L	R	Adherent
RERF-LC-MS	24.780643	0.927903	10	Resistant	Y	Y	Y	Y	Y	LUAD	MSS/MSI-L	D/F12	Adherent
NCI-H1666	23.95053	0.917101	10	Resistant	Y	Y	Y	Y	Y	LUAD	MSS/MSI-L	D/F12	Semi-Adherent
HCC-78	22.826237	0.913729	10	Resistant	Y	Y	Y	Y	Y	LUAD	MSS/MSI-L	R	Adherent
NCI-H650	21.514047	0.898667	10	Resistant	Y	Y	Y	Y	Y	LUAD	MSS/MSI-L	D/F12	Adherent
NCI-H2405	20.606829	0.912524	10	Resistant	Y	Y	Y	Y	Y	LUAD	MSS/MSI-L	D/F12	Adherent
EMC-BAC-1	19.834582	0.902333	10	Resistant	Y	Y	Y	Y	Y	LUAD	MSS/MSI-L	R	Adherent

NCI-H2085	19.704721	0.915862	10	Resistant	Y	Y	Y	Y	Y	LUAD	MSS/MSI-L	D/F12	Adherent
NCI-H838	17.337305	0.90705	10	Resistant	Y	Y	Y	Y	Y	LUAD	MSS/MSI-L	R	Adherent
NCI-H1793	17.047637	0.856084	10	Resistant	Y	Y	Y	Y	Y	LUAD	MSS/MSI-L	D/F12	Adherent
LXF-289	16.498515	0.913907	10	Resistant	Y	Y	Y	Y	Y	LUAD	MSS/MSI-L	D/F12	Adherent
RERF-LC-KJ	16.293029	0.898284	10	Resistant	Y	Y	Y	Y	Y	LUAD	MSS/MSI-L	R	Adherent
NCI-H2087	15.102645	0.899371	10	Resistant	Y	Y	Y	Y	Y	LUAD	MSS/MSI-L	D/F12	Semi-Adherent
NCI-H1437	14.939191	0.89956	10	Resistant	Y	Y	N	Y	Y	LUAD	MSS/MSI-L	R	Adherent
NCI-H1944	13.146397	0.890639	10	Resistant	Y	Y	Y	Y	Y	LUAD	MSS/MSI-L	D/F12	Adherent
NCI-H2228	12.832075	0.846224	10	Resistant	Y	Y	Y	Y	Y	LUAD	MSS/MSI-L	R	Adherent
NCI-H2122	12.402294	0.891954	10	Resistant	Y	Y	Y	Y	Y	LUAD	MSS/MSI-L	R	Adherent
NCI-H1792	10.624647	0.904862	10	Resistant	Y	Y	Y	Y	Y	LUAD	MSS/MSI-L	R	Adherent
NCI-H2030	10.403689	0.865021	10	Resistant	Y	Y	Y	Y	Y	LUAD	MSS/MSI-L	R	Adherent
A549	9.920681	0.842218	10	Sensitive	Y	Y	Y	Y	Y	LUAD	MSS/MSI-L	D/F12	Adherent
LC-2-ad	9.840459	0.871995	10	Sensitive	Y	Y	Y	Y	Y	LUAD	MSS/MSI-L	R	Adherent
COR-L105	9.531665	0.825541	10	Sensitive	Y	Y	Y	Y	Y	LUAD	MSS/MSI-L	R	Adherent
HCC-827	9.042716	0.834263	10	Sensitive	Y	Y	Y	Y	Y	LUAD	MSS/MSI-L	R	Adherent
PC-14	8.871175	0.865356	10	Sensitive	Y	Y	Y	Y	Y	LUAD	MSS/MSI-L	R	Adherent
SK-LU-1	8.620789	0.83209	10	Sensitive	Y	Y	Y	Y	Y	LUAD	MSS/MSI-L	D/F12	Adherent
EMC-BAC-2	8.424787	0.845288	10	Sensitive	Y	Y	Y	Y	Y	LUAD	MSS/MSI-L	R	Adherent
NCI-H1734	7.617296	0.839165	10	Sensitive	Y	Y	Y	Y	Y	LUAD	MSS/MSI-L	R	Adherent
NCI-H1651	7.606348	0.845173	10	Sensitive	Y	Y	Y	Y	Y	LUAD	MSS/MSI-L	D/F12	Adherent
NCI-H1650	6.950768	0.797451	10	Sensitive	Y	Y	Y	Y	Y	LUAD	MSS/MSI-L	R	Adherent
NCI-H2023	6.66373	0.814015	10	Sensitive	Y	Y	Y	Y	Y	LUAD	MSS/MSI-L	R	Adherent
NCI-H1355	6.34353	0.788441	10	Sensitive	Y	Y	Y	Y	Y	LUAD	MSS/MSI-L	D/F12	Adherent
201T	5.693731	0.795563	10	Sensitive	Y	Y	Y	Y	Y	LUAD	MSS/MSI-L	R	Adherent
NCI-H23	5.522476	0.821406	10	Sensitive	Y	Y	Y	Y	Y	LUAD	MSS/MSI-L	R	Adherent
NCI-H1781	5.08645	0.803819	10	Sensitive	Y	Y	Y	Y	Y	LUAD	MSS/MSI-L	R	Adherent
HOP-62	4.918779	0.798173	10	Sensitive	Y	Y	Y	N	Y	LUAD	MSS/MSI-L	R	Adherent
ABC-1	2.749392	0.709317	10	Sensitive	Y	Y	Y	Y	Y	LUAD	MSS/MSI-L	D/F12	Adherent
HCC-44	2.555889	0.722004	10	Sensitive	Y	Y	Y	Y	Y	LUAD	MSS/MSI-L	R	Adherent
