# Supplementary material for: Identification of Mutations Related to Cisplatin-Resistance and Prognosis of Patients With Lung Adenocarcinoma
Source: Front Pharmacol. 2020 Oct 29;11:572627. doi: 10.3389/fphar.2020.572627 (PMC7658917; doi:10.3389/fphar.2020.572627)
Supplement: Supplementary file 3 [file Table2_v1.pdf]

Supplementary Table2. The mutations of GDSC-LUAD cell lines associated with cisplatin response.

gene	Pval	Wildtype (Mean: Cisplatin IC50)	Mutation (Mean: Cisplatin IC50)	Mutation vs Wildtype
XIRP2	7.88E-05	21.71589969	92.98736176	Cisplatin Resistant
SLIT3	0.003586641	40.75458659	131.3058365	Cisplatin Resistant
TMPRSS15	0.003756438	41.73069931	135.7273241	Cisplatin Resistant
DDX60L	0.003913043	40.60249354	171.2868043	Cisplatin Resistant
PTPRD	0.004877808	46.70393657	100.9641516	Cisplatin Resistant
LPHN2	0.005572206	44.99660678	137.3893593	Cisplatin Resistant
RAPGEF6	0.010674734	41.98151696	145.8152353	Cisplatin Resistant
VAV3	0.014082452	49.22854159	104.743005	Cisplatin Resistant
EVC2	0.014993949	48.97571815	106.6933573	Cisplatin Resistant
F8	0.014993949	53.32628439	73.13184629	Cisplatin Resistant
ZNF804A	0.015292547	50.85172002	79.810456	Cisplatin Resistant
ZNF804B	0.015309336	44.78926979	127.2138728	Cisplatin Resistant
CXorf59	0.015957023	46.08565098	128.9881611	Cisplatin Resistant
ADAMTS20	0.016132906	46.14484537	103.8155167	Cisplatin Resistant
ACACB	0.016233991	42.97868719	139.2089825	Cisplatin Resistant
PRUNE2	0.016322324	40.20510817	107.2787284	Cisplatin Resistant
SCN1A	0.020374199	53.71814111	70.10895157	Cisplatin Resistant
ABCB11	0.021631046	47.12520464	111.7383044	Cisplatin Resistant
FAM135B	0.02488901	44.33231471	101.604905	Cisplatin

				Resistant
FAT4	0.025171559	51.09789336	70.71009236	Cisplatin Resistant
KIAA1409	0.02621845	42.198479	116.5107574	Cisplatin Resistant
UGT2A1	0.027366937	61.23224881	12.14297786	Cisplatin Sensitive
FSTL5	0.027510174	41.8879189	117.9223942	Cisplatin Resistant
NBEAL1	0.031914552	47.58771627	101.8867817	Cisplatin Resistant
GRIN2B	0.035335213	44.51951334	129.0010093	Cisplatin Resistant
CDH23	0.036746493	45.362479	80.05309356	Cisplatin Resistant
VWA5B1	0.040575016	49.41475493	103.3065021	Cisplatin Resistant
LOC340578	0.043483673	48.67358583	101.480279	Cisplatin Resistant
AFF2	0.045732843	49.5331567	83.17131327	Cisplatin Resistant
GREB1	0.048120443	49.26314458	97.57445225	Cisplatin Resistant
